# The effects of glycemic index on prostate cancer progression in a xenograft mouse model

**DOI:** 10.1038/s41391-023-00769-w

**Published:** 2023-12-11

**Authors:** Gloria Cecilia Galván, Everardo Macias, Sergio Sanders, Adela Ramirez-Torres, Shannon Stock, Sungyong You, Celine E. Riera, Patrick Tamukong, Stephanie A. Smith-Warner, Jeanine M. Genkinger, Daniel J. Luthringer, Michael R. Freeman, Stephen J. Freedland

**Affiliations:** 1 Department of Urology, Cedars-Sinai Medical Center, Los Angeles, CA USA; 2 Department of Pathology, Duke University School of Medicine, Durham, NC USA; 3 Department of Mathematics and Computer Science, College of the Holy Cross, Worcester, MA USA; 4 Department of Surgery, Urology Section, Veterans Affairs Health Care System, Durham, NC USA; 5 Department of Computational Biomedicine, Cedars-Sinai Medical Center, Los Angeles, CA USA; 6 Department of Biomedical Sciences, Cedars-Sinai Medical Center, Los Angeles, CA USA; 7 Department of Neurology, Cedars-Sinai Medical Center, Los Angeles, CA USA; 8 Departments of Nutrition and Epidemiology, Harvard T.H. Chan School of Public Health, Boston, MA USA; 9 Mailman School of Public Health, Columbia University, New York, NY USA; 10 Herbert Irving Comprehensive Cancer Center, Columbia University, New York, NY USA; 11 Department of Pathology, Cedars-Sinai Medical Center, Los Angeles, CA USA

**Keywords:** Prostate cancer, Cancer therapy

## Abstract

**Background:**

Previously, we found low-carbohydrate diets slowed prostate cancer (PC) growth and increased survival vs. a Western diet in mice, by inhibiting the insulin/IGF-1 axis. Thus, we tested whether modifying carbohydrate *quality* to lower glycemic index (GI) without changing *quantity* results in similar benefits as with reduced *quantity*.

**Methods:**

Male SCID mice injected with LAPC-4 cells were single-housed and randomized when their tumors reached 200 mm^3^ on average to a LoGI (48% carbohydrate kcal, from Hylon-VII) or HiGI Western diet (48% carbohydrate kcal, from sucrose). Body weight and tumor volume were measured weekly. Body composition was assessed 35 days after randomization. Blood glucose and serum insulin, IGF-1 and IGFBP3 were measured at study end when tumor volumes reached 800 mm^3^. We analyzed gene expression of mice tumors by RNA-sequencing and human tumors using the Prostate Cancer Transcriptome Atlas.

**Results:**

There were no significant differences in tumor volume (*P* > 0.05), tumor proliferation (*P* = 0.29), and overall survival (*P* = 0.15) between groups. At 35 days after randomization, the LoGI group had 30% lower body fat (*P* = 0.007) despite similar body weight (*P* = 0.58). At sacrifice, LoGI mice had smaller livers (*P* < 0.001) and lower glucose (*P* = 0.15), insulin (*P* = 0.11), IGF-1 (*P* = 0.07) and IGF-1:IGFBP3 ratio (*P* = 0.05), and higher IGFBP3 (*P* = 0.09) vs. HiGI, although none of these metabolic differences reached statistical significance. We observed differential gene expression and pathway enrichment in mice tumors by diet. The most upregulated and downregulated gene in the LoGI group showed expression patterns more closely resembling expression in human benign prostate tissue vs. PC.

**Conclusions:**

In this single mouse xenograft model, consuming a low GI diet did not delay PC growth or survival vs. a high GI diet despite suggestions of decreased activation of the insulin/IGF-1 pathway. These data suggest that improving carbohydrate quality alone while consuming a high carbohydrate diet may not effectively slow PC growth.

## Introduction

Prostate cancer (PC) is a major public health concern [1]. While multiple local and systemic therapies are available, they can cause significant side effects and are not always curative. Therefore, alternative therapies are needed. Given their low toxicity, potential to improve overall health, and ability to be self-implemented, there is strong interest in dietary approaches for PC management.

Carbohydrates are one main energy source in the American diet. Carbohydrate intake leads to insulin secretion, which activates proliferation and survival pathways known to promote cancer development and growth [2]. In previous xenograft studies, we showed carbohydrate reduction slowed PC growth and increased survival. Versus mice fed an isocaloric Western diet (44% kcal of carbohydrates), mice fed a no-carbohydrate diet had prolonged survival, smaller tumors, reduced insulin, and increased insulin-like growth factor-binding protein 3 (IGFBP3), which has tumor suppressing activity [3]. Another animal study showed consuming a low-carbohydrate diet (20% kcal of carbohydrates) yielded similar results to a no-carbohydrate diet [4]. However, long-term adherence to no-carbohydrate or low-carbohydrate diets could be difficult for some, especially cancer patients who are facing challenges from both having cancer and undergoing treatment. Therefore, we tested if analogous to reducing carbohydrate *quantity*, whether modifying carbohydrate *quality* also impacted PC growth.

Glycemic index (GI) assesses carbohydrate quality by determining the impact of carbohydrates on blood glucose [5]. Low GI foods result in slower rise of glucose and insulin levels. Given the role of glucose and insulin in promoting tumor growth, we hypothesized a low GI diet may slow tumor growth. Studies testing the association between GI and PC risk in humans have shown mixed findings and to date [6–9], there are no animal studies testing a low GI diet for PC. We investigated the effect of carbohydrate quality on PC growth and survival in a xenograft model comparing high vs. low GI Western diets.

## Materials and methods

### Cell culture

To model early-stage PC, we used a human hormone-sensitive PC cell line with wild-type androgen receptor shown to respond to dietary modulation [3, 4]. Los Angeles Prostate Cancer 4 (LAPC-4) cells were obtained from Dr. William Aronson from UCLA [10]. Cells were cultured in Iscove’s modified medium containing with 10% fetal bovine serum supplemented with 1 nM of synthetic androgen R1881 and maintained in 5% CO_2_ at 37 °C and harvested after trypsinization when they reached ~80% confluency in log-phase growth.

### Xenograft study

To determine the effects of carbohydrate quality on PC growth and survival, we conducted a xenograft mouse study comparing two diets with different GI. Male 6–8-week-old severe combined immunodeficient (CB.17 scid/scid) mice (*n* = 64) were purchased from Taconic Biosciences, Inc (Germantown, NY) after obtaining approval from Cedars-Sinai Medical Center Institutional Animal Care and Use Committee (protocol IACUC006821). After an acclimation period, they were fed an *ad libitum* HiGI diet at day 1 of the study and housed at 5 mice/cage. On day 14, all mice were injected with 0.15 ml of a 1:1 solution of 5 × 10^^5^ LAPC-4 cells and Matrigel (Corning, Corning, NY) into the lower right flank. Tumor volumes, once palpable, were measured weekly using digital calipers and calculated using the formula: width × height × length × 0.5236. When tumor volumes reached 200 mm^3^ on average, mice were single-housed and randomized to a diet (*n* = 32 mice/group): HiGI or LoGI Western diet provided ad libitum. Investigators, blinded to group allocation, randomized mice by tumor size to ensure similar tumor volumes across groups. Diets were purchased from Research Diets Inc (New Brunswick, NJ) and are described in Table 1. Diet composition was nearly identical except for different predominant source of carbohydrates: sucrose for the HiGI and Hylon-VII (70% amylose, 30% amylopectin) for the LoGI. Body weight was monitored every week. Mice were sacrificed by euthanasia with CO_2_ after a four-hour fast when their tumor volume reached 800 mm^3^. Blood was obtained via cardiac puncture. Tumors and livers were harvested and weighed. One half of each tumor was snap-frozen with liquid nitrogen while the other half was placed in 10% neutral buffered formalin and subsequently embedded in paraffin blocks. The health of one mouse was compromised and therefore was euthanized soon after randomization, per Cedars-Sinai institutional criteria. The mouse belonged to the LoGI group; however, the health deterioration did not seem related to the diet and was excluded from analyses.

**Table 1:** Composition of experimental diets.

	HiGI WD		LoGI WD	
Kcal/g	4.5		3.8	
	%gm	%kcal	%gm	%kcal
Protein	20	18	17	18
Carbohydrate	53	48	60	48
Fat	17	35	15	35
**Total**		**100**		**100**
**Ingredient**	**gm**	**kcal**	**gm**	**kcal**
Casein	195	780	195	780
DL-Methionine	3	12	3	12
Hylon VII (70% amylose, 30% amylopectin)	0	0	571	1599
Maltodextrin	0	0	125	500
Sucrose	524.66	2099	0	0
Cellulose, BW200	50	0	50	0
Milk Fat, Anhydrous	83	747	83	747
Lard	83	747	83	747
Corn Oil	8.3	75	8.3	75
Mineral Mix S10001	35	0	35	0
Calcium Carbonate	4	0	4	0
Vitamin Mix V10001	10	40	10	40
Choline Bitartrate	2	0	2	0
Cholesterol	1.5	0	1.5	0
Ethoxyquin	0.04	0	0.04	0
Total	1000	4499	1171.34	4500

### Body composition analysis

Mouse body composition was determined using the Echo-MRI^TM^ system (Echo-MRI, Houston, TX), which uses quantitative magnetic resonance technology to precisely measure body fat, lean and water mass. Measurements along with body weight were taken 35 days after randomization.

### Glucose, insulin, IGF-1, IGFBP-3 measurements

Blood glucose was measured at sacrifice using Accu-Chek Aviva Plus glucose strips and meter (Roche Diabetes Care Inc, Indianapolis, IN). Serum was obtained by centrifugation of whole blood lysates after allowed to clot. Serum insulin, IGF-1 and IGFBP-3 were determined by enzyme-linked immunoassays (ELISA) kits per manufacturer’s instructions from Rat/Mouse Insulin (Millipore Sigma, Burlington, MA), Mouse/Rat IGF-1 (R&D Systems, Minneapolis, MN) and Mouse/Rat IGFBP-3 (ALPCO, Salem, NH) from a subset of mice (*n* = 11/group).

### Tumor proliferation measurement and histology review

Tumor proliferation was assessed by immunohistochemistry in 4 µm sections from FFPE tumor blocks at Cedar-Sinai Biobank and Research Pathology Resource. Slides were incubated with Ki67 monoclonal antibody (Roche Diagnostics, Ventana Medical Systems, Oro Valley, AZ) for 32 min at 37 °C. Quantification was done by a single investigator blinded to the study groups using an automated imaging software. Percent of positive cells was determined from cells presenting weak, medium and strong signals out of the total cells in the sample. To determine histological differences, sections from tumor blocks were stained with hematoxylin and eosin (H&E), and reviewed by a board-certified pathologist in a blinded fashion.

### RNA sequencing and gene expression analysis

Tumors from a subset of mice (*n* = 3/group) were used for gene expression analysis by RNA-sequencing at Cedars-Sinai’s Applied Genomics Computation and Translational Shared Resource. Total RNA was isolated with Direct-Zol RNA isolation kit (Zymo Research Corporation, Irvine, CA). RNA quantity and quality were assessed using QuBit Fluorometer (ThermoFisher Scientific, Waltham, MA) and 2100 Bioanalyzer (Agilent, Santa Clara, CA), prior to sequencing on the Illumina NovaSeq 6000 (Illumina, San Diego, CA). Data quality was assessed using MultiQC software [11]. Given tumors were xenografts (i.e., human cells), and thus have human cell expression patterns, we first used BBMap [12] to map reads to human (GENCODE human genome: Release 38(GRCh38.p13)) and mouse (MOUSE Genome: Release M22(GRCm38.p6)) genome and removed reads unique to mouse alone. Second, we used an established STAR-RSEM mapping and quantification pipeline for read alignment to the human genome and transcript quantification [13–15]. Count normalization and differential gene expression analysis were performed using the edgeR software using Benjamini & Hochberg to adjust for multiple comparisons [16]. Differentially expressed genes were selected at false discovery rate < 0.05 and log2 fold change (FC) |≥1 | . Differential enrichment of transcriptomic pathways was determined by ranking all analyzed genes by FC in Pre-ranked Gene Set Enrichment Analysis (GSEA) [17] with hallmark gene sets from the Molecular Signatures Database hallmark gene set collection (MSigDB) [18]. We used the GSEA Desktop version 4.1.0. Hallmark pathways enriched at nominal *p*-value < 0.05 were defined as significant.

To determine association of gene expression with human disease course, we used the online tool Prostate Cancer Transcriptome Atlas (PCTA; thepcta.org), which includes data from 1321 specimens from 38 human PC cohorts [19]. We determined gene expression by disease course: benign, primary PC (Gleason scores <7, 7, and >7), and metastatic castration-resistant PC (mCRPC). Differences between subsets were determined by one-way ANOVA and Rank-sum test.

### Statistical analysis

We calculated sample size for 80% power at a two-sided significance level of 0.05 to detect differences of at least 0.36 mm^3 between groups at the final endpoint. Shapiro Wilk test was used to test for normality of the data. F-test of equality of variances was used to test variance between groups. Assumptions of data independence, normality and variance were considered when choosing statistical tests. Two-sample *t t*est or Wilcoxon rank sum exact test was used to compare groups, as appropriate. Survival was defined as time from randomization to sacrifice and is shown graphically by Kaplan–Meier curves. Differences in survival between groups were tested using a log-rank test. Statistical significance was defined by a *p*-value of *P* < 0.05. Statistical analyses were performed using Stata (version 14.2) and R (version 4.0.4).

## Results

### Diet did not impact survival or tumor growth

**Fig. 1:**
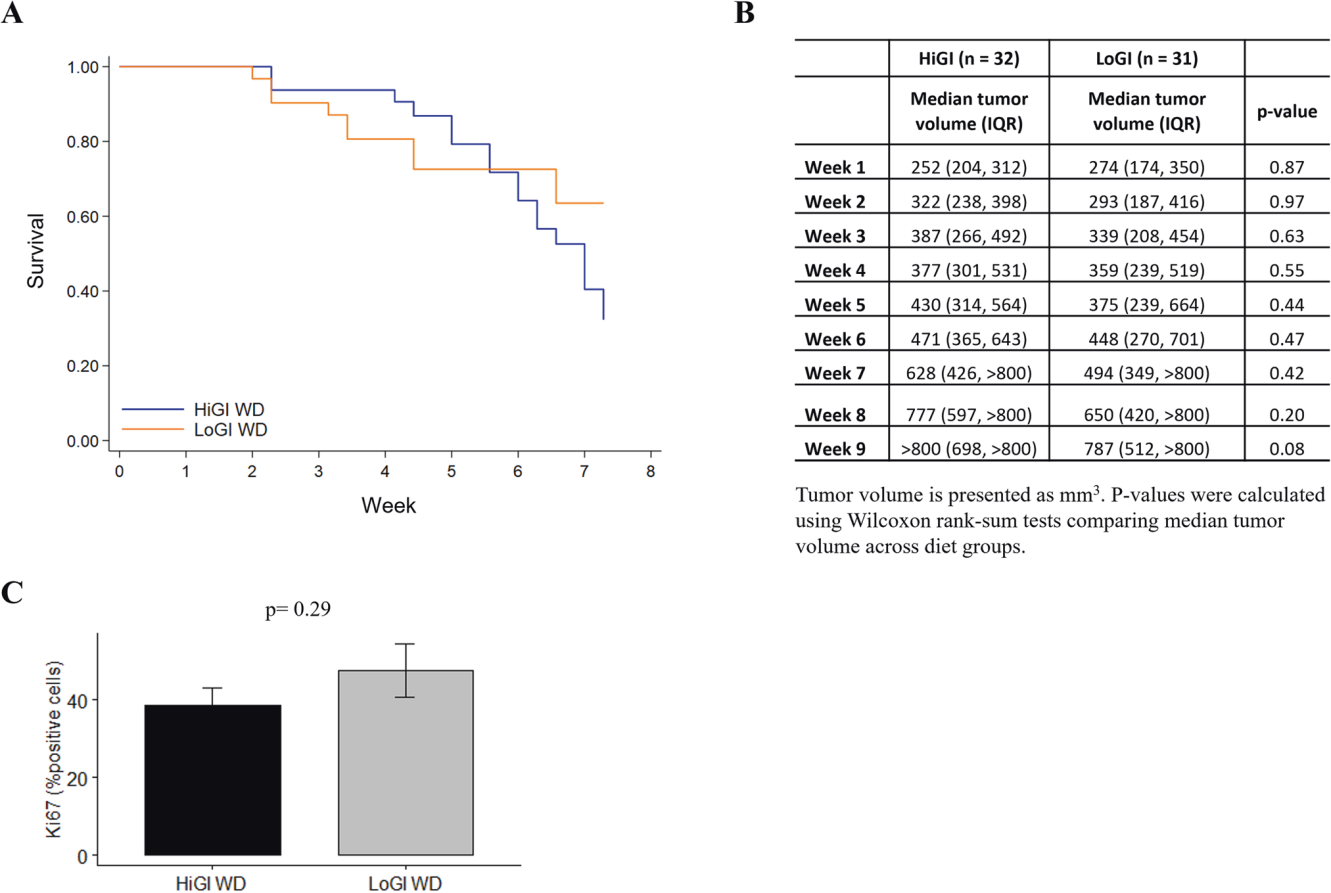
Impact of glycemic index on survival and tumor growth in mice with PC. **A** Kaplan–Meier survival curves for survival stratified by group. Differences between groups were tested using a log-rank test. **B** Summary statistics for tumor volume stratified by diet. P-values were calculated using Wilcoxon rank-sum tests comparing median tumor volume across diet groups. **C** Quantification of tumor Ki67 expression detected by immunohistochemistry. Values are presented as the mean of each group. Error bars represent standard deviation. Mean comparison was done by Two sample *t* test.

We found no difference in overall survival (time to 800 mm^3^ tumor volume) (*P* = 0.15, Fig. 1A) and no differences in median tumor volume at any point of the study (all *P* > 0.05, Fig. 1B) between diet groups. There was no difference between diets in tumor weight at sacrifice (*P* = 0.20, data not shown) or in Ki67 staining in the tumor (*P* = 0.29, Fig. 1C). Additionally, we did not observe any histological differences between groups.

### Mice fed a LoGI diet had lower body fat and liver weight

**Fig. 2:**
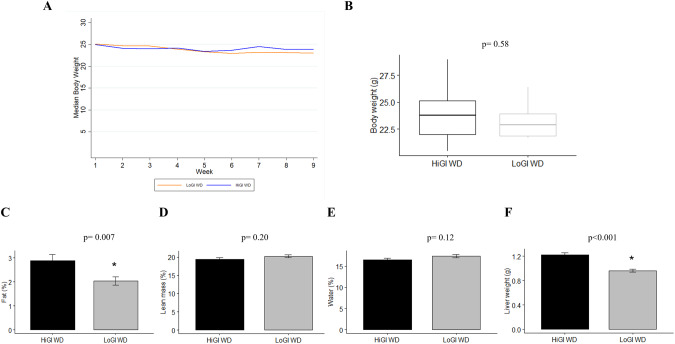
Effect of experimental diets on body weight, body composition and liver weight. **A** Mice were weighed twice per week. Values are presented as median body weight of each group after being randomized to their diet. **B**–**E** Body weight and body composition on day 35 after diet randomization. Body composition was determined using quantitative magnetic resonance technology. **F** Livers from mice were weighed at sacrifice. In bar graphs (**C**–**F**), values are presented as the mean of each group. Error bars represent standard deviation. Two sample *t* t*e*st or Wilcoxon rank-sum test were used for group comparison (*P < 0.05).

Body weight was similar between groups throughout the study (Fig. 2A), but body composition at 35 days after randomization differed between groups (Fig. 2C–E). Mice fed a LoGI diet had 30% lower percent body fat vs those fed a HiGI diet (*P* = 0.007, Fig. 2C), while maintaining the same body weight (*P* = 0.58, Fig. 2B). Correspondingly, percent lean and water mass were similar among diet groups (Fig. 2D, E). Liver weight at sacrifice was 21% lower for mice fed a LoGI diet vs a HiGI diet (*P* < 0.001, Fig. 2F).

### A LoGI diet altered the insulin/IGF-1 axis

**Fig. 3:**
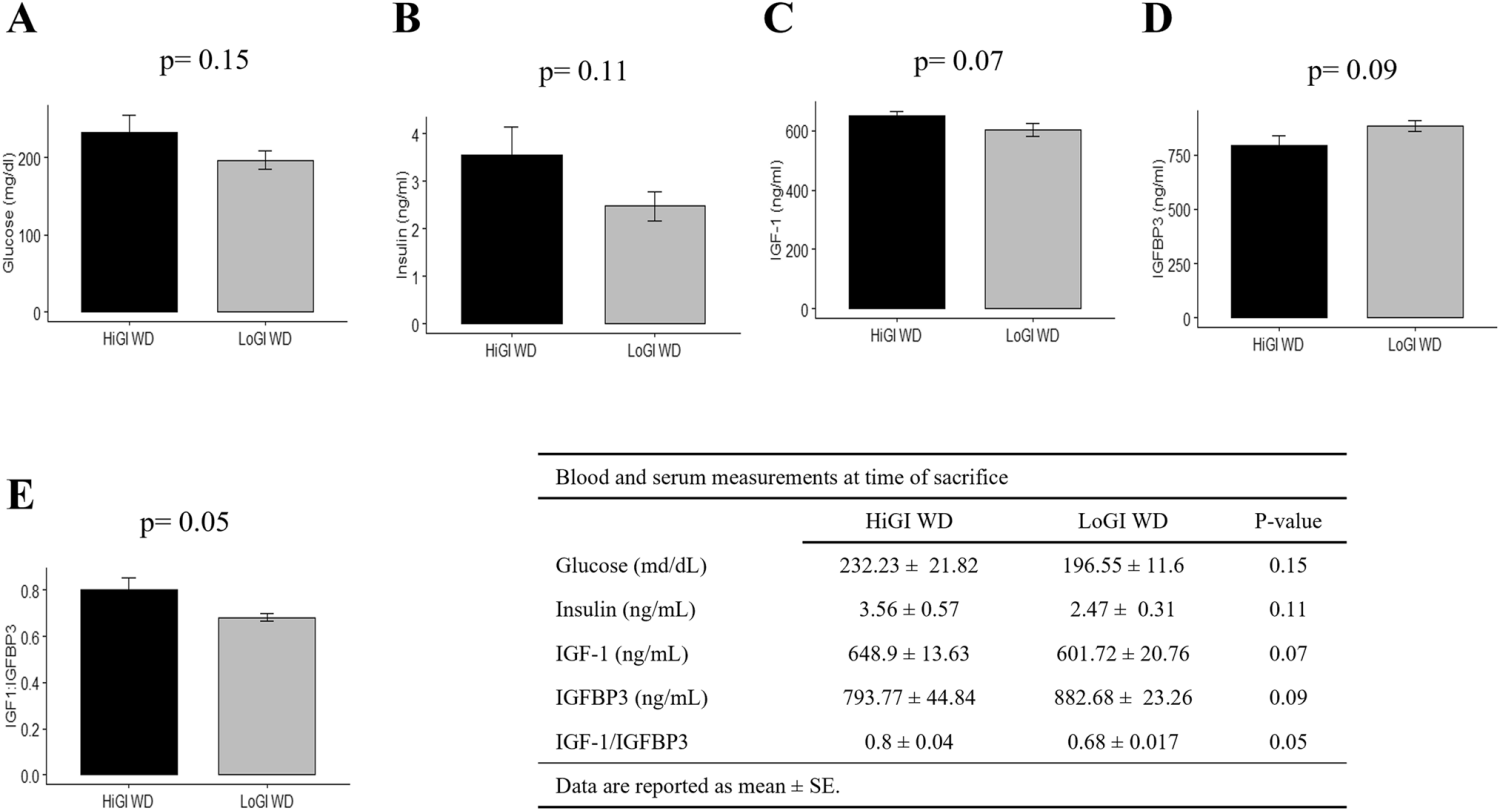
Detection of markers of the insulin/IGF-1 pathway in mice at sacrifice. **A** Blood glucose levels. **B**–**E** Serum levels of insulin, IGF-1, IGFBP3 and IGF-1:IGFBP3 ratio. Values are presented as the mean of each group. Error bars represent standard deviation. Mean comparison was done by Two sample *t* test.

Although not statistically significant, consistent trends of lower insulin/IGF-1 axis measures were seen for the LoGI vs the HiGI group (Fig. 3). Mice fed a LoGI diet had lower blood glucose (*P* = 0.15) and serum insulin (*P* = 0.11), IGF-1 (*P* = 0.07) and IGF-1:IGFBP3 (*P* = 0.05), and higher IGFBP3 (*P* = 0.09), vs the HiGI group (Fig. 3A–E).

### GI had an impact on gene expression

**Fig. 4:**
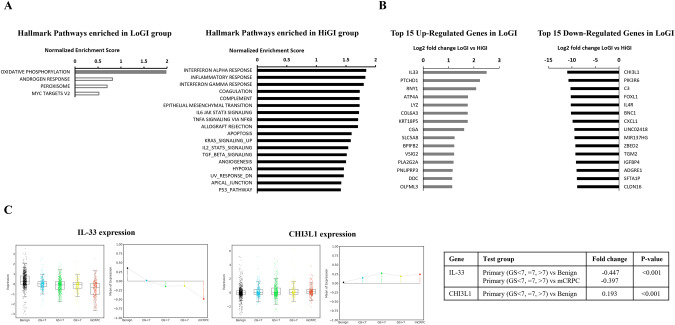
Gene expression analysis. **A** Top pathways enriched in each diet group in mice tumors. Solid bars represent significantly enriched pathways. **B** Top 15 differentially expressed genes by diet group in mice tumors. **C** Expression of differentially expressed genes IL-33 and CHI3L1 in human PC by disease course.

Tumor RNA-sequencing data were analyzed to determine pathway enrichment and differential gene expression by group. We found 18 pathways significantly upregulated in the HiGI group (i.e., downregulated in LoGI), while only one pathway was significantly upregulated in the LoGI group (Fig. 4A). The top 15 differentially expressed genes by diet are graphed in Fig. 4B. To determine their relevance in human PC, we selected the top downregulated and upregulated genes in the LoGI vs. HiGI group and determined their expression by disease course in the PCTA. Expression of IL-33 (interleukin-33), the top upregulated gene in the LoGI group (FC = 2.5), is lower in primary PC and mCRPC vs benign prostate tissue (*P* < 0.001), while expression of CHI3L1 (chitinase-3-like protein-1), the top downregulated gene in the LoGI group (FC = −10.98) is higher in primary PC vs benign prostate tissue (*P* < 0.001) (Fig. 4C).

## Discussion

Dietary interventions, particularly those including carbohydrate modulation, are not a new concept in cancer research. Still, most of the research to date focused on carbohydrate restriction, instead of modifying carbohydrate *quality*. Human studies show a potential benefit to lowering GI in our diets for cancer prevention and overall health, however, the extent of this modification, the mechanisms behind those effects and the association with cancer progression remain unknown [6, 20]. In this study, we aimed to determine the impact of consuming a high or low GI diet on PC progression using a xenograft model.

Previous research shows low-carbohydrate diets (i.e., reducing carbohydrate *quantity*) delayed PC growth and improved survival [3, 4]. Meanwhile, our current results suggest that modifying the types of carbohydrates consumed (i.e., altering carbohydrate *quality)*, while keeping carbohydrate intake high and the same across groups, was not enough to delay PC growth and improve survival. However, our study design did not exactly mimic a human eating pattern, given that mice are nocturnal and eat in smaller amounts continuously rather than in big meals. This is a limitation of the study that could have reduced the pro-cancerous effects of a high GI. Importantly, we saw no detrimental effects on PC progression or survival from consuming a LoGI diet, but rather found favorable impacts on body composition and suggestive benefits on glucose homeostasis, suggesting a LoGI diet may have overall health benefits.

Our goal was to compare the impact of diet composition on tumor growth independent of weight loss. For this reason, body weight in the mice was carefully monitored and remained the same between groups throughout the study. Still, mice fed a LoGI diet had 30% lower percent body fat (*P* = 0.007) than those on a HiGI diet. Due to the methodology (Echo-MRI^TM^) we used to determine body composition, we were only able to quantify total body fat mass but unable to identify adipose tissue distribution in the body. However, we do not anticipate this observation was related to cachexia, a state commonly observed in advanced cancer stages, known to change body composition and induce wasting and weight loss because the LoGI group had non-significantly lower tumor volumes at this time point compared to the HiGI group. Also, there were no differences in body weight between groups at any point of the study, and no weight or muscle mass loss. In sum, these findings show GI impacts body composition. Intriguingly, a systematic review of human studies found a low GI or low glycemic load diet was associated with reduced body weight and total fat mass [21]. These studies focused on people who were overweight or obese, while we saw an impact on fat mass in non-obese mice without weight loss. Altogether, these findings suggest modulating carbohydrate quality is an effective way of improving body composition, though ultimately the impact of this on cancer outcomes remains unclear.

When liver weights were taken at sacrifice, livers from mice fed a LoGI diet weighed >20% less than those fed a HiGI diet (*P* < 0.001). This is a relevant finding as high liver weight could be an indication of fat infiltration. In a randomized controlled trial testing macronutrient quality on liver fat content, a low GI/low saturated fat diet resulted in significantly lower glycemic response and hepatic fat, compared to a high GI/high saturated fat diet [22]. Similarly, improved glucose control, independent of body weight change, is associated with reduced non-alcoholic fatty liver disease [23]. Although further research is needed to confirm if improving glycemic control through a low GI diet results in protection from hepatic steatosis, if true, this result would be a highly impactful finding given the negative health consequences of fatty liver.

We hypothesized that consuming a low GI diet would inhibit tumor growth by decreasing the activation of the insulin/IGF-1 signaling axis compared to a high GI diet. To test this, we measured blood glucose and serum levels of insulin, IGF-1, and IGFBP-3. Mice fed a LoGI diet had suggestively lower levels of glucose, insulin, IGF-1, and IGF-1: IGFBP-3 ratio and higher IGFBP3 levels than the group fed a HiGI diet. Although none of these comparisons reached statistical significance, there was a clear trend suggesting a favorable impact of consuming a low GI diet on the insulin/IGF-1 pathway. Inhibition of IGF-1 in PC has been associated with slower PC growth in multiple animal and in vitro studies [24, 25]. Also, IGFBP-3, one of IGF-1 binding proteins, is thought to have tumor suppressive effects independent of IGF-1, by promoting apoptosis, and inhibiting growth, invasion, and angiogenesis [26–28]. Although our study may have had insufficient power to examine these associations, as these measurements were done only on a subset of mice, our results suggest that consuming a LoGI diet may not inhibit the insulin/IGF-1 pathway strongly enough to impact tumor growth.

To further understand the impact of GI, we analyzed the PC transcriptomes from mice to determine effects on pathways aside from the insulin/IGF-1 pathway. We identified 18 pathways upregulated in the HiGI group. Intriguingly, more than half of these were immune or inflammation related, including interferon alpha and interferon gamma response, inflammatory response, and TNFa via NFkB, IL6/Jak/Stat3, IL2/Stat5 and TGF-beta signaling pathways. Others included epithelial mesenchymal transition, apoptosis, angiogenesis, hypoxia, and KRAS and P53 signaling, which are related to cancer processes, such as cell proliferation and metastatic potential. Meanwhile, the oxidative phosphorylation pathway was upregulated in the LoGI group. A potential hypothesis to explain this is that tumors in the LoGI group may be shifting to beta-oxidation to get energy from fat breakdown instead of sugar, which could also explain why this group had a lower percent body fat. If true, this raises the possibility that in tumor types that are more glucose dependent (i.e., PCs are typically not considered highly glucose dependent tumors), such as some glioblastoma subtypes, anti-tumor activity could be seen, though this would require further study [29]. Surprisingly and contrary to our expectations, we did not see differential enrichment of the PI3K/mTOR pathway in this analysis. This finding, along with the modest reduction in insulin and IGF-1 levels, supports the idea that modulation of GI alone when consuming a high carbohydrate diet does not impact pro-growth pathways strongly enough to impact tumor volume, especially compared to carbohydrate restriction.

The top upregulated gene in the LoGI group was IL-33, a cytokine currently being studied as a potential therapy for immune homeostasis and for its role in cancer immune-surveillance [30, 31]. According to our analysis from >1300 PC human specimens in the PCTA database, IL-33 expression was significantly higher in benign prostate tissue compared to primary tumors and even lowest in mCRPC (*P* < 0.001). A previous study showed low IL-33 levels in metastatic vs. primary tumors and its association with higher PC progression and recurrence [31]. In contrast, the top downregulated gene in the LoGI group was CHI3L1, which is associated with cancer cell proliferation, invasion, metastasis, and angiogenesis and is highly expressed in metastatic PC [32, 33]. Furthermore, it may contribute to an immunosuppressive tumor microenvironment by activating tumor-associated macrophages and Th2 polarization of CD4^+^ T-cells [34]. Combined with the enrichment of immune-related pathways mentioned above, these findings suggest GI perturbation could impact immune functions in cancer. In our analysis of human PC, CHI3L1 gene expression was higher in PC vs benign prostate tissue (*P* < 0.001). In summary, intriguingly, a LoGI diet induced gene expression changes (upregulated IL-33 and downregulated CHI3L1) that mirror expression patterns of benign prostate tissue more than PC, suggesting a LoGI diet may be associated with PC prevention. However, interpretation of these results should be done cautiously since results from previous human cohorts did not consistently see an association between a low GI diet and cancer risk. In a study from France, investigators did find an association between low GI food and beverages and lower cancer risk. This was true for overall cancer, breast cancer and postmenopausal breast cancer [20]. However, specific to PC, published research shows mixed findings. High GI was associated with increased PC risk in a case-control study performed in Iranian men [6] and a 2019 dose-response meta-analysis [6, 9]. Meanwhile, in the Prostate, Lung, Colorectal and Ovarian Cancer Screening Trial cohort, authors did not find an association between PC incidence and GI but stated their ability to detect associations was limited by having a narrow GI range in the cohort [8]. Similarly, there was no association found between GI and PC risk in two meta-analyses [7, 35]. Ultimately, further validation among different populations is needed. Thus, we consider the results from our gene expression analysis to be hypothesis-generating. Further studies are needed to provide additional information about how carbohydrate quality modulates cancer cell signaling and to determine the impact of these genes/pathways on long-term outcomes.

While consuming a LoGI Western diet did not improve survival vs a HiGI Western diet in our PC xenograft study, we were limited to one model and therefore were unable to generalize our conclusions to PC progression and survival. Given the literature on GI and PC shows mixed results and is limited to PC risk and not progression, further research is needed to better identify the role of GI in PC progression. Future studies considering modifications of carbohydrate quality while also modifying carbohydrate quantity and their effects on PC growth and survival are needed, for example using glycemic load, which considers GI and carbohydrate amount, instead of GI only.

## Conclusion

Although consuming a low GI high carbohydrate diet was not enough to delay PC growth in mice, compared to a high GI high carbohydrate diet, findings from this study show eating a low GI diet can have a positive impact on body composition, specifically by reducing adiposity. Furthermore, a LoGI diet reduced expression of pro-tumor and inflammation-related genes, while promoting a healthier metabolic state by improving glucose homeostasis, though these latter findings were not statistically significant. Ultimately, further research is needed to gain additional insight on the role of GI in PC outcomes and to determine the effects of GI when consuming a low carbohydrate diet.

## Data Availability

The RNA-sequencing datasets generated during the current study have been deposited in NCBI’s Gene Expression Omnibus [36] and are available through the GEO Series accession number GSE246780 (https://www.ncbi.nlm.nih.gov/geo/query/acc.cgi?acc=GSE246780).
